# Postoperative around-the-clock administration of intravenous acetaminophen for pain control following robot-assisted radical prostatectomy

**DOI:** 10.1038/s41598-021-84866-7

**Published:** 2021-03-04

**Authors:** Shogo Inoue, Hirotsugu Miyoshi, Keisuke Hieda, Tetsutaro Hayashi, Yasuo M. Tsutsumi, Jun Teishima

**Affiliations:** 1 Department of Urology, Hiroshima University Graduate School of Biomedical Sciences, 1-2-3 Kasumi, Minami-ku, Hiroshima, 734-8551 Japan; 2 Department of Anesthesiology and Critical Care, Hiroshima University Hospital, Hiroshima, Japan

**Keywords:** Signs and symptoms, Urology

## Abstract

The objective of this study was to examine the impact of around-the-clock (ATC) administration of intravenous (IV) acetaminophen following robot-assisted radical prostatectomy (RARP). Intravenous infusion of acetaminophen was started on the day of the operation at 1000 mg/dose every 6 h, and the infusion was continued on a fixed schedule until postoperative day 2 a.m. In a retrospective observational study, we compared 127 patients who were administered IV acetaminophen on a fixed schedule (ATC group) with 485 patients who were administered analgesic drugs only as needed (PRN group). We investigated postoperative pain intensity and additional analgesic consumption on postoperative day 0, 1, 2, 3, and 5 between the two groups. Postoperative pain scores were significantly lower in the ATC group than in the PRN group at 1 and 2 days, and this period matched the duration of ATC administration of IV acetaminophen. Postoperative frequency of rescue analgesia was significantly lower in the ATC group than in the PRN group at postoperative 0, 1, and 2 days. ATC administration of IV acetaminophen has the potential to be a very versatile and valuable additional dose to achieve appropriate postoperative analgesia in patients with RARP.

## Introduction

Robot-assisted radical prostatectomy (RARP) is known to cause less postoperative pain than open surgery. However, during the period immediately after surgery, patients have reported experiencing moderate to severe pain following RARP^1,2^. Although the incision size is small, visceral irritation and prolonged pneumoperitoneum due to high pressure can increase postoperative pain after RARP^2^. In addition, insertion of a urethral catheter is thought to be mandatory for several days after radical prostatectomy, but penile pain and bladder spasms associated with the urethral catheter become issues in postoperative pain management^3^. Despite how common the procedure has become, there is little objective data on postoperative pain and convalescence after RARP.

These characteristics of pain after RARP suggest that a scheduled dosing regimen may be effective. However, many clinicians fear that regular scheduled administration of analgesics may result in unnecessary and/or excessive administration of analgesics, resulting in undesirable and potentially harmful side effects^4^. A randomized clinical trial (RCT) evaluated the efficacy of around-the-clock (ATC) administration of analgesics in patients after RARP^5^. This RCT showed that there was no difference in pain score or opioid use between patients receiving intravenous (IV) acetaminophen and IV placebo in the perioperative setting of RARP.

Acetaminophen is a widely accepted standard treatment option for mild acute postoperative pain. IV acetaminophen is currently approved by the Food and Drug Administration (FDA) for analgesic indications. It is a fast and effective drug with fewer side effects compared with non-steroidal anti-inflammatory drug (NSAID) which cause gastrointestinal bleeding and renal insufficiency. These drug properties of acetaminophen are suitable for pain control in patients following RARP, which is common procedure on elderly patients. Surprisingly, the use of IV acetaminophen has not been studied in patients following radical prostatectomy; no outcomes are currently available^5^.

Due to the lack of studies on the effectiveness of ATC administration of analgesics for the postoperative pain management in patients following RARP, additional study is needed. We hypothesized that patients who received regularly scheduled IV acetaminophen after RARP would report less pain than those who received analgesics as needed (pro re nata; PRN). The purpose of this study was thus to determine whether ATC administration of IV acetaminophen increased or decreased pain intensity and increased or decreased frequency of rescue analgesia in patients who underwent RARP compared with PRN administration. In addition, we evaluated the effect of ATC administration on the severity of catheter related bladder discomfort (CRBD).

## Patients and methods

**Figure 1:**
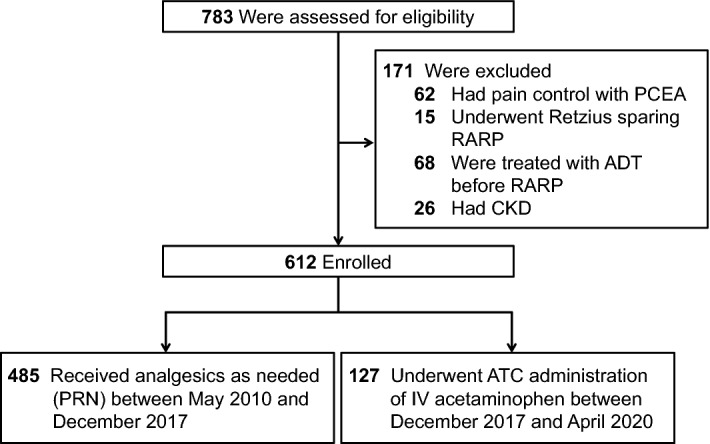
Flowchart for study inclusion among patients with localized PCa who had undergone RARP. *PCEA* patient-controlled epidural analgesia, *RARP* robot-assisted radical prostatectomy, *ADT* androgen deprivation therapy, *CKD* chronic kidney disease, *ATC* around-the-clock, *PRN* pro re nata, *PCa* prostate cancer.

A total of 783 patients with localized PCa who had undergone RARP at Hiroshima University Hospital between May 2010 and April 2020 were included in this study. Exclusion criteria included chronic opioid use, liver disease, allergy to acetaminophen, baseline dementia, chronic diathesis, and chronic kidney disease. We excluded 62 patients who had pain control with patient-controlled epidural analgesia (PCEA), 15 patients who underwent Retzius sparing RARP, 68 patients who were treated with Androgen deprivation therapy (ADT) before RARP, and 26 patients who had chronic kidney disease (CKD). Opioid analgesics were not used in all patients for postoperative pain control following RARP in this cohort. A new postsurgical analgesia protocol (ATC administration) has been applied to all patients following RARP since December 2017. We compared 127 patients who underwent ATC administration of IV acetaminophen (ATC group) with 485 patients under PRN (PRN group). This is a retrospective observational study. After obtaining the approval of institutional review board of Hiroshima University Hospital, the medical records of all patients with PRN group (between May 2010 and December 2017) and patients with ATC group (between December 2017 and April 2020) were retrospectively reviewed (Fig. 1).

In all patients, intraoperative anesthesia was managed under general anesthesia with tracheal intubation. Of the patients who met the indication criteria for epidural anesthesia, an epidural catheter was inserted only in those who wished it to be combined with general anesthesia. General anesthesia was induced with remifentanil at 0.25–0.5 µg/kg/min, propofol at 2.0–3.0 µg/ml with a target-controlled infusion system (TCI pump TE-371, Terumo Corporation, Japan) and 6–10 mg/kg of rocuronium, and was maintained with propofol at 1.0–3.5 µg/ml of TCI, sevoflurane at 1.0–2.0% or desflurane at 3–5% in combination with remifentanil at 0.05–0.5 µg/kg/min. Doses of the anesthetics were determined with reference to the electroencephalogram monitor Entropy (GE Healthcare, Helsinki, Finland). Fentanyl, acetaminophen (1000 mg or 15 mg/kg), and flurbiprofen (50 mg) were administered in the operating room at the discretion of the anesthesiologist.

In the ATC group, IV infusion of acetaminophen (Acelio Intravenous Injection; TERUMO Co. Ltd., Tokyo, Japan) was started every 6 h on the day of the operation at 1000 mg/dose for patients weighing ≥ 50 kg or 15 mg per kg of the body weight for patients weighing < 50 kg. These administrations continued on a fixed schedule until postoperative day 2 a.m. after the start of oral intake and oral medications. In the case of PRN administration of analgesics, all patients received on-demand analgesic drugs, such as diclofenac sodium suppository (50 mg) or IV flurbiprofen axetil (50 mg) on fasting and loxoprofen sodium (60 mg) or Celecoxib (200 mg) after the start of oral intake and oral medication. Narcotics were not routinely prescribed in our protocol of pain management.

The primary endpoint was postoperative pain intensity measured on a numeric rating scale (NRS) that ranged from 0 = “no pain” to 10 = “unbearable pain” on days 0, 1, 2, 3, and 5 postoperatively. Pain scores were recorded at scheduled times until discharge. Postoperative pain level was measured on the NRS by a nurse once every 8 h during routine measurements of vital signs. Postoperative pain intensity and additional analgesic consumption (number of doses) were compared between the two groups on days 0, 1, 2, 3, and 5 postoperatively. The CRBD score was rated on a 4-point scale and defined as follows: 0 = no discomfort, 1 = mild discomfort, 2 = moderate discomfort, 3 = severe discomfort. CRBD score was measured by a nurse in the morning on postoperative day 0, 1, 2, 3, and 5 until removal of the urethral catheter.

### Statistical analysis

The baseline patient characteristics and surgical outcomes were examined using a chi-squared test, Mann–Whitney U test, and one-way analysis of variance (ANOVA) analysis. In all tests, a *p* value of < 0.05 was determined to be statistically significant. All values reported are means ± standard deviation, and the survey data were analysed using an unpaired t-test. They were also analysed using JMP 15 software (SAS Institute, Cary, NC, USA).

### Ethical approval

All procedures performed in studies involving human participants were in accordance with the ethical standards of the institutional and/or national research committee and with the 1964 Helsinki declaration and its later amendments or comparable ethical standards. The Institutional Review Board (IRB) of Hiroshima University Hospital approved this research protocol (IRB No. 3786).

### Consent to participate

This was a retrospective observational study, and the requirement for written informed consent was waived by the IRB. The research content was posted on outpatient of Hiroshima University Hospital after approval and data of cases without opt-out intention were adopted.

## Results

### Demographic characteristics

Table 1 summarizes the details of the baseline patient characteristics and surgical outcomes. The mean age of the enrolled patients was significantly higher in the ATC group than in the PRN group (68.1 ± 6.2 vs. 66.6 ± 6.1 years, *p* = 0.015). There were no significant differences between the ATC and PRN groups in the mean preoperative eGFR value. The rate of pelvic lymph node dissection was significantly higher in the ATC group than in the PRN group (33 vs. 22%, *p* = 0.008), and operation time was significantly longer in the ATC group than in the PRN group (238.1 ± 60.1 vs. 201.0 ± 50.6 min, *p* = 0.001). In the distribution of the D’Amico risk group classification, the ratio of high risk was significantly higher in the ATC than in the PRN group (40% vs. 31%, *p* = 0.029). There was a significant difference between groups in analgesics at the end of anesthesia; however, there was no significant difference in intraoperative dose of Fentanyl. In the evaluation of postoperative outcomes, there were no significant differences between groups in time to ambulation, oral intake, and length of stay.

**Table 1:** Patient characteristics.

Characteristics	ATC	RPN	*p* value
Case, n (%)	127 (21)	485 (79)	
Age, mean (years)	68.1 ± 6.2	66.6 ± 6.1	0.015
BMI, mean (kg/m^2^)	23.5 ± 2.9	23.4 ± 2.7	0.690
PSA, mean (ng/ml)	9.8 ± 7.5	9.0 ± 6.6	0.230
**D’Amico risk group, n (%)**			0.029
Low	10 (8)	68 (14)	
Intermediate	66 (52)	266 (55)	
High	51 (40)	151 (31)	
eGFR, mean (ml/min)	69.1 ± 12.3	71.4 ± 12.7	0.059
Pelvic lymph node dissection, n (%)	42 (33)	105 (22)	0.008
Resected volume, mean (g)	43.1 ± 15.9	40.4 ± 13.9	0.064
Operation time, mean (min)	238.1 ± 60.1	201.0 ± 50.6	0.001
Insufflation time, mean (min)	203.0 ± 56.7	167.7 ± 49.0	0.001
EBL, mean (ml)	157.2 ± 107.5	177.5 ± 178.7	0.223
Intraoperative dose of Fentanyl (μg)	312.5 ± 138.5	288.5 ± 143.0	0.093
**Analgesics at the end of anesthesia, n (%)**			0.001
Acetaminophen	63 (50)	75 (15)	
NSAIDs	43 (34)	338 (70)	
Both	10 (8)	6 (1)	
None	11 (9)	66 (14)	
Time to ambulation (day)	1.00 ± 0.00	1.01 ± 0.23	0.611
Time to oral intake (day)	1.03 ± 0.36	1.06 ± 0.58	0.661
Time to catheterization (day)	6.70 ± 1.32	6.44 ± 1.91	0.149
Length of stay (day)	9.85 ± 2.21	10.13 ± 2.61	0.269

*BMI* body mass index, *PSA* prostate specific antigen, *EBL* estimated blood loss, *ATC* around-the-clock, *PRN* pro re nata.

### Longitudinal evaluation of postoperative pain scores (Fig. 2)

**Figure 2:**
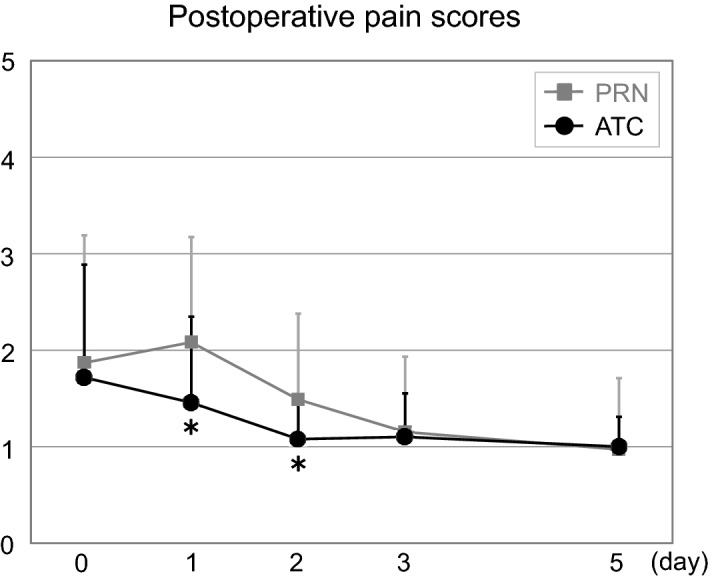
Comparison of postoperative pain scores after surgery. *ATC* around-the-clock, *PRN* pro re nata, *p < 0.05 versus baseline value.

Postoperative pain score (0–10) data were collected from 612 patients, among whom 127 patients (21%) were in the ATC group. Postoperative pain scores were significantly lower in the ATC group than in the PRN group at postoperative 1 (1.46 ± 0.89 vs. 2.08 ± 1.09, *p* = 0.001) and 2 (1.08 ± 0.37 vs. 1.49 ± 0.89, *p* = 0.001) days, and this period matched the duration of ATC administration of IV acetaminophen (Fig. 2).

### Longitudinal evaluation of frequency of rescue analgesia (Fig. 3)

**Figure 3:**
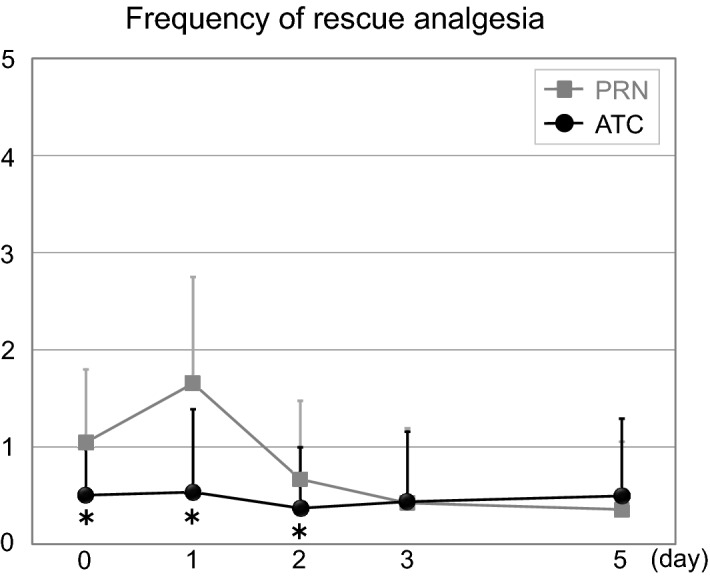
Comparison of frequency of rescue analgesia after surgery. *ATC* around-the-clock, *PRN* pro re nata, *p < 0.05 versus baseline value.

Postoperative frequency of rescue analgesia was significantly lower in the ATC group than in the PRN group at postoperative 0 (0.50 ± 0.60 vs. 1.05 ± 0.75, *p* = 0.001), 1 (0.54 ± 0.85 vs. 1.65 ± 1.10, *p* = 0.001), 2 (0.37 ± 0.63 vs. 0.67 ± 0.81, *p* = 0.001) days, and this period matched the period of ATC administration of IV acetaminophen (Fig. 3).

### Longitudinal evaluation of CRBD scores (Fig. 4)

**Figure 4:**
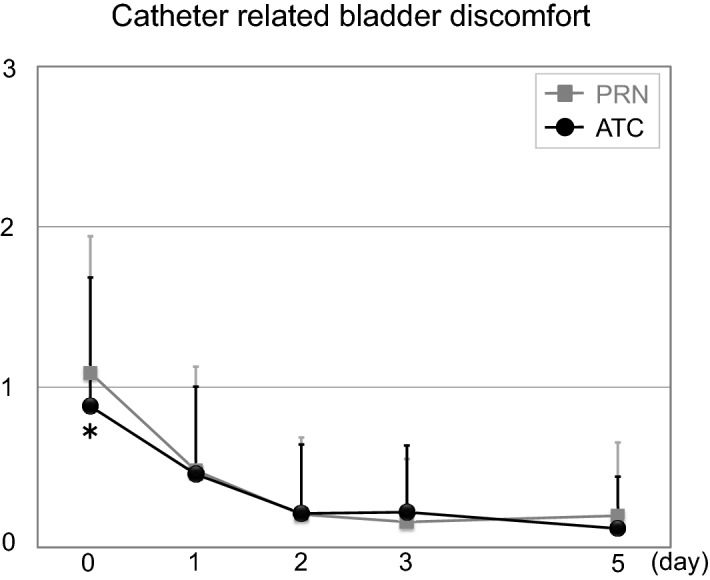
Comparison of catheter related bladder discomfort after surgery. *ATC* around-the-clock, *PRN* pro re nata, *p < 0.05 versus baseline value.

CRBD scores (0–3) were significantly lower in the ATC group than in the PRN group at postoperative 0 (0.88 ± 0.80 vs. 1.09 ± 0.85, *p* = 0.014) days. There were no significant differences between groups at postoperative 1 (0.46 ± 0.55 vs. 0.48 ± 0.65, *p* = 0.705), 2 (0.21 ± 0.43 vs. 0.21 ± 0.48, *p* = 0.891), 3 (0.22 ± 0.42 vs. 0.16 ± 0.39, *p* = 0.120), and 5 (0.12 ± 0.32 vs. 0.20 ± 0.46, *p* = 0.065) days (Fig. 4). Average duration of catheterization was 6.51 days; as a result, CRBD was mostly relieved at the end of catheterization without administration of analgesics.

## Discussion

Prostate cancer can be managed by a variety of strategies, including extirpative and ablative surgery, radiation therapy, or even conservative methods that include active surveillance in selected low-risk patients. Given the relative lack of definite data to suggest cancer-specific outcome differences in certain groups of patients with localized prostate cancer treated by any of the above-mentioned methods, patients and their physicians are left with a variety of secondary factors to consider when making a choice between the available treatment options for their prostate cancer^6^. For patients, one of the most important factors in weighing prostatectomy against other treatment strategies is the potential postoperative pain and convalescence associated with surgery^3^.

Within the broad category of extirpative surgery, there are a variety of approaches to radical prostatectomy, including open prostatectomy, which can be performed via a retropubic or perineal approach, laparoscopic prostatectomy, or RARP. RARP has become popular because of its advantages such as three-dimensional visualization of the surgical field and shortening of the learning curve^7^. In addition, the incision length in RARP is rather short and postoperative pain is assumed to be minimal. While proponents of RARP suggest a variety of advantages to using a robot for pelvic surgery, including reduced blood loss, reduced transfusion requirements, improved dexterity of instruments, one of the main advantages appears to be decreased convalescence and reduced postoperative pain^8–10^.

One of the proposed benefits of adoptation of the da Vinci Surgical System for RARP is decreased postoperative pain compared with open radical prostatectomy, but there is limited prospective data on what a patient undergoing RARP might reasonably expect in terms of pain or analgesic requirements immediately after surgery and in the first week after recovering from the operation^11^. However, RARP has been reported to cause postoperative pain comparable to that of open prostatectomy in the immediate postoperative period^1,12^. Appropriate analgesia is needed after RARP to manage acute postoperative pain and minimize complications associated with narcotics use. However, the current literature only includes the use of opioids to treat pain in the perioperative setting of RARP^2^; only a small trial used oral acetaminophen as a measure for analgesia in RARP^13^.

Pain control during the perioperative period has focused on nonopioid analgesics instead of opioids, whose use is associated with many side effects such as ileus, respiratory compromise, delirium, hyperalgesia, urinary retention, and addiction potential^14–17^. Various perioperative multimodal anesthesia have emerged in the light of the nationwide focus on reducing opioid use to combat the opioid epidemic^13,18^. Multimodal analgesic strategies are effective in reducing these complications and accelerating patient recovery and discharge^19^. It is well known that postoperative stress and pain can cause hypoxia, fluid overload, immobilization, gastrointestinal paralysis, and fatigue^20^. These factors are interdependent and can be significantly influenced by postoperative management.

IV acetaminophen, which inhibits prostaglandin synthesis, has been approved by the US FDA for management of mild to moderate pain, management of moderate to severe pain with opioid analgesics, and reduction of fever in adults and children over 2 years of age^17,21^. Opioids such as fentanyl, hydrocodone, morphine, and oxycodone have been used perioperatively to reduce postoperative pain and discomfort^22,23^. Receiving nonopioid therapy would be effective not only in the perioperative period but also in the long term after surgery. Therefore, it not only avoids the side effects of opioids, but also reduces the overall cost of patient care by shortening the recovery time and length of stay in hospital^22^. IV acetaminophen is a fast and effective drug that can support the effects of opioids and has fewer side effects caused by NSAIDs such as gastrointestinal bleeding and renal dysfunction. Recently, several benefits of using IV acetaminophen in the perioperative setting have been shown to reduce overall opiate consumption in patients, improve analgesia, and reduce postoperative nausea and vomiting^14,24–26^. IV acetaminophen has been shown in several studies to be more advantageous than the oral form in the perioperative setting^27,28^. As a result, it is considered to be very versatile and safe for the elderly, with adequate postoperative analgesia having been achieved in patients undergoing a wide variety of surgical procedures and in many hospital settings.

Although IV acetaminophen has been studied in urologic surgery^24,29^, its use in prostatectomies has not been a focus and few studies on it are available. RARP is one of the most common procedures performed in men, and the results of the current study show the use of perioperative IV acetaminophen to be an ideal pain management for the patients after RARP.

Our hypothesis is that ATC administration of IV acetaminophen improves postoperative pain scores and reduce the number of on-demand analgesic drugs. We examined the effect of IV acetaminophen on ATC administration; in particular, we investigated pain scores and the number of rescue analgesics after RARP. The results suggest that ATC administration of IV acetaminophen was more effective than PRN administration in reducing pain intensity and frequency of rescue analgesics in the first two days following RARP. In addition, we encountered poor pain control when using conventional PRN dosing of analgesics and problems of unexpected and additional usage of postoperative analgesics. Given the predictable properties of postoperative pain after RARP, ATC administration of analgesics should be considered during the early postoperative period, while PRN administration should be considered once the patient's pain intensity and analgesic requirements decrease.

Urinary catheter insertion may cause various degrees of catheter-related bladder discomfort (CRBD) during the postoperative period in patients who have underwent surgical procedures, especially urinary intervention. CRBD symptoms associated with indwelling urinary catheters are similar to those of overactive bladder, including suprapubic discomfort, urinary urgency, pollakiuria, and a burning sensation^30,31^. This study evaluated the efficacy of ATC administration of IV acetaminophen in patients who underwent RARP and urinary catheterization for CRBD intensity. We found that intraoperative ATC administration of IV acetaminophen significantly decreased the severity of CRBD more than PRN administration did only at postoperative day 0.

We feel this information is valuable in counseling patients about their treatment options after diagnosis of localized prostate cancer, because the anxiety of potential pain associated with surgery is certainly a factor that weighs into a patient’s decision-making process. We think this study will give physicians an objective tool in counseling patients who are considering undergoing RARP but have reasonable concerns about what they may expect during their periods of convalescence. This information should be useful in counseling patients on the merits of RARP relative to other less invasive therapies for prostate cancer^3^.

There were some limitations in our study. First, this is a single center study of only the Japanese population, so it may not be applicable to other populations. We recommend more large-scale, well-designed RCTs on patients following RARP to improve confidence in our conclusions. Second, we investigated postoperative frequency of rescue analgesia for the evaluation of ATC administration. The rescue analgesics were different in our protocol, so we should improve the study protocol for a more accurate evaluation. Third, we did not evaluate side effects such as liver disfunction. Many drugs including analgesics were used during the perioperative period, and it was unclear whether any of the side effects were particular to acetaminophen. Therefore, we did not investigate complications in this study population.

In summary, the results of this study suggest the effectiveness of ATC dosing of acetaminophen during early postoperative recovery of patients following RARP, particularly in the first 2 days. Further research may be needed to determine if ATC administration benefits other populations following urologic surgery.

## Conclusion

ATC administration of IV acetaminophen has the potential to be a very versatile and valuable additional dose to achieve appropriate postoperative analgesia in patients with RARP. We feel this information will be valuable in counseling patients about their treatment options after diagnosis of localized prostate cancer, because the anxiety of potential pain associated with surgery is certainly a factor that weighs into a patient’s decision-making process.
